# MicroRNA Expression in Bovine Cumulus Cells in Relation to Oocyte Quality

**DOI:** 10.3390/ncrna3010012

**Published:** 2017-03-11

**Authors:** Karen Uhde, Helena T. A. van Tol, Tom A. E. Stout, Bernard A. J. Roelen

**Affiliations:** 1 Department of Farm Animal Health, Faculty of Veterinary Medicine, Utrecht University, Yalelaan 104, 3584CM Utrecht, The Netherlands; K.Uhde@uu.nl (K.U.); H.T.A.vanTol@uu.nl (H.T.A.T.); T.A.E.Stout@uu.nl (T.A.E.S.); 2 Department of Equine Sciences, Faculty of Veterinary Medicine, Utrecht University, Yalelaan 112, 3584CM Utrecht, The Netherlands

**Keywords:** microRNA, cumulus cells, oocyte quality

## Abstract

Cumulus cells play an essential role during oocyte maturation and the acquisition of fertilizability and developmental competence. Micro(mi)RNAs can post-transcriptionally regulate mRNA expression, and we hypothesized that miRNA profiles in cumulus cells could serve as an indicator of oocyte quality. Cumulus cell biopsies from cumulus−oocyte−complexes that either yielded a blastocyst or failed to cleave after exposure to sperm cells were analyzed for miRNA expression. On average, 332 miRNA species with more than 10 reads and 240 miRNA species with more than 50 reads were identified in cumulus cells; this included nine previously undescribed microRNAs. The most highly expressed miRNAs in cumulus cells were miR-21, members of the let-7 family and miR-155. However, no repeatable differences in miRNA expression between the cumulus cells from oocytes that became blastocysts versus those from non-cleaved oocytes were identified. Further examination of individual cumulus cell samples showed a wide variability in miRNA expression level. We therefore conclude that miRNA expression in cumulus cells cannot be used as an oocyte quality marker.

## 1. Introduction

Mammalian oocytes acquire the capacity to become fertilized and acquire developmental competence during their final maturation within the ovary. These oocytes originate from female primordial germ cells that proliferated while migrating towards the gonadal ridges and continue to proliferate until gonadal sex differentiation. The cells are classified as oocytes from the moment they stop dividing and enter meiosis [1]. Meiosis in developing oocytes arrests at the first prophase, and depending on the species, this meiotic arrest can be maintained for decades [2]. As early as this time point, the oocyte acquires competence to develop into an embryo after fertilization [3]. The oocyte only resumes meiosis shortly before ovulation, in response to luteinizing hormone stimulation. After the resumption of meiosis and in addition to chromosome segregation, the oocyte needs to redistribute organelles and proteins in a process known as cytoplasmic maturation [4]. For these processes, the oocyte relies on molecules present within the follicular fluid and on intimate contact with the somatic cells that surround the oocyte, known as cumulus cells. Together, they form the cumulus−oocyte−complex (COC). Tightly-regulated communication between the oocyte and surrounding cumulus cells is important at various stages, including oocyte growth, maturation and fertilization [5,6,7]. Exactly how the cumulus cells contribute to the fertilizability and developmental competence of the oocyte, hereafter referred to as oocyte quality, is not known. From in vitro maturation and fertilization experiments, it has however become clear that large differences exist between the quality of oocytes, and it seems likely that cumulus cells are at least partly responsible for the oocyte quality.

Cumulus cells are connected to each other and to the oocyte by direct cell-cell contact. Exchange of small (<1 kDa) molecules, like cyclic adenosine monophosphate (cAMP), is thought to occur through gap junctions [8,9,10], whereas the passage of larger molecules, including RNA, can occur via zonula-adherens-like junctions [11,12]. Communication between the oocyte and cumulus cells is bidirectional [13], with the cumulus cells providing the oocyte with factors and signaling molecules [6], but the oocyte also sending signals to the cumulus cells [9]. If communication between the cumulus cells and the oocyte is disturbed, oocyte quality can be affected, which can in turn compromise subsequent embryo development [14]. Indeed, in vitro maturation of oocytes in the absence of cumulus cells markedly reduces the developmental competence of oocytes [15].

For human in vitro fertilization (IVF) in particular, it would be beneficial if fertilizable and developmentally-competent oocytes could be identified before fertilization, without damaging the oocyte. Oocyte morphology has been demonstrated to be a poor predictor of quality and is rather subjective [16,17]. A molecular marker to predict oocyte quality before fertilization would improve embryo development and pregnancy rates and could be used to reduce the number of surplus embryos produced and the resulting ethical conflict regarding the ultimate fate of these embryos [18]. One useful model animal for human oocyte development is the cow since, like man, it is a mono-ovulatory species; in addition, it has a comparable time course of oogenesis [19,20]. Furthermore, the blastocyst development rate after in vitro fertilization in cattle is ~35%, comparable with that obtained in human IVF programs [21,22]. Not surprisingly, efforts have been undertaken to identify non-invasive quality markers for oocytes, including analysis of gene expression in cumulus cells [23,24,25,26,27] and investigation of the composition of follicular fluid [25,28].

MicroRNAs (miRNAs) are highly conserved, small (21–25 nucleotides in length), non-coding RNAs that can regulate gene expression [29,30]. Via sequence complementarity, the seed sequences of miRNAs can bind to the 3’UTR of target mRNAs and, in combination with an Argonaute family protein, the miRNA can regulate the expression of the target gene by degrading that mRNA or inhibiting its translation. It has been suggested that a single miRNA species can regulate ~100 different target genes [31], enabling a broad level of regulation.

miRNAs are processed from longer transcripts by the RNase III-like enzymes Dicer and Drosha. Genetic deletion of Dicer results in embryonic lethality in mice [32], and when Dicer was specifically deleted in growing oocytes, they failed to cleave after fertilization, indicating the importance of miRNAs to oocyte competence [32]. Apart from expression in oocytes, miRNAs have also been detected in cumulus cells [14,33], and it has been demonstrated that the miRNA expression profiles of both oocyte and cumulus cells change during maturation [33,34,35,36,37]. Identification of differentially-expressed miRNAs in corona radiata cells and the outer cumulus cells from human oocytes indicated a role for these miRNAs in nutritional and regulatory signaling between oocytes and cumulus cells [38].

In this study, we examined the miRNA expression profiles of bovine cumulus cells and compared those from COCs that yielded blastocyst after fertilization versus those that remained non-cleaved after exposure to sperm.

## 2. Results

### 2.1. Comparison of Individual versus Group Culture

In order to identify miRNA species expressed in cumulus cells that could potentially predict quality of the enclosed oocyte, cumulus biopsies were obtained from COCs after in vitro maturation. Subsequent individual fertilization of COCs and individual embryo culture were performed to follow the progression of each oocyte individually and thereby allow correlation of the cumulus miRNA expression profile to oocyte quality, retrospectively. First, to analyze whether individual embryo culture affected the efficiency of blastocyst formation, embryos were cultured either individually, but with shared culture medium, or in groups of 30–50. The percentages of oocytes that cleaved or developed into blastocysts were similar for the two culture conditions (Table 1), demonstrating the validity of the single embryo culture system.

### 2.2. Sequencing of miRNAs in Cumulus Cells

In order to determine whether the miRNA expression profiles of cumulus biopsies from COCs that contained fertilizable and developmentally-competent oocytes (i.e., yielded a blastocyst) differed from those that contained oocytes of poor quality, cells were harvested for small RNA sequencing. After COCs had been matured, a biopsy of the cumulus cells was obtained, and the COCs were cultured individually for fertilization and embryo development.

**Figure 1:**
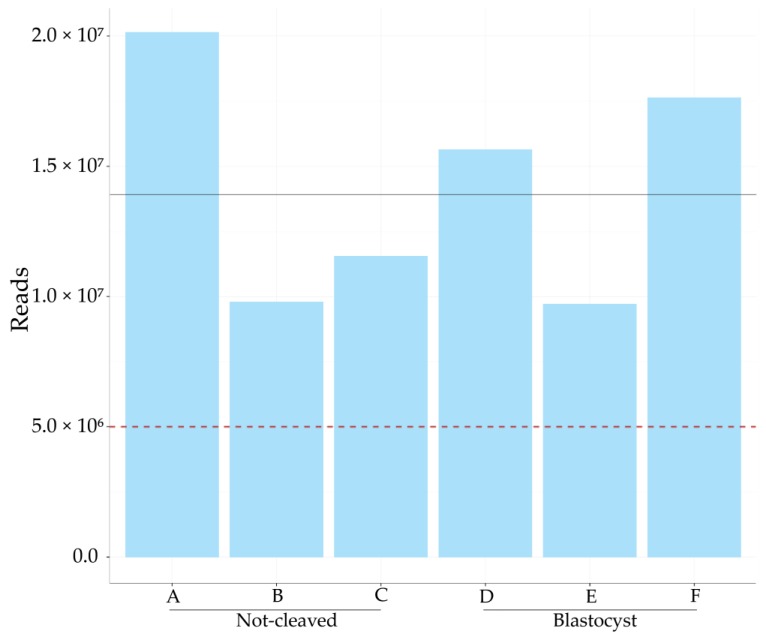
Total number of micro RNA (miRNA) reads in each sample. The total number of miRNA sized reads for each sample of cumulus cells pooled from 10 to 16 bovine cumulus–oocyte complexes; on average, 14 million reads were obtained per sample, as indicated by the black line. The red dotted line marks five million reads, the amount considered sufficient for meaningful analysis. A, B, C = cumulus pieces surrounding oocytes that failed to cleave after exposure to sperm; D, E, F = cumulus pieces surrounding oocytes that developed into a blastocyst after fertilization.

Cumulus cells surrounding an oocyte that (1) developed into a blastocyst or (2) failed to cleave after exposure to sperm were pooled to ensure sufficient amounts of RNA (each pooled sample contained cumulus cells from 10 to 16 individual COCs). Three pooled samples of cumulus cells harvested from oocytes that did not cleave (referred to as A, B and C) and three pooled samples of cumulus cells that had enclosed oocytes that developed into blastocysts (D, E and F) were sequenced for small RNAs; they yielded an average of 14 million reads (Figure 1).

**Figure 2:**
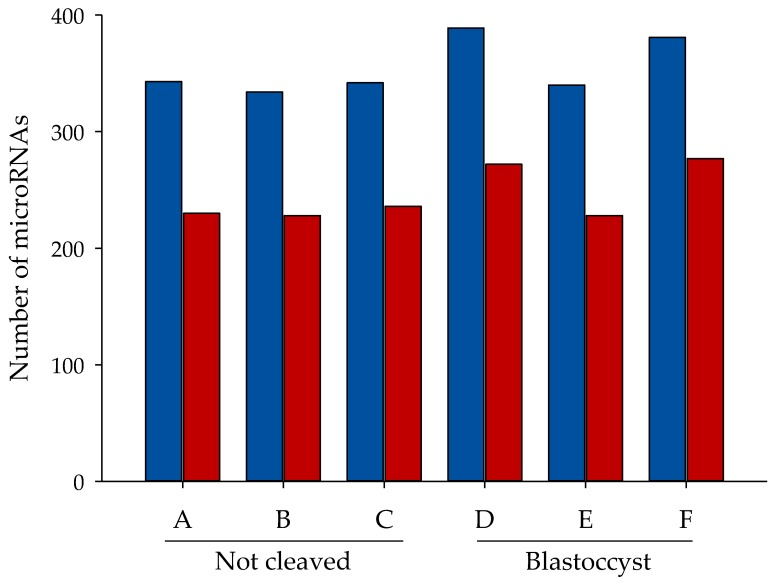
Number of miRNA reads. Number of known miRNAs with more than a given number of mapped reads: >0, but <50 reads per sample for blue bars and >50 per sample for red bars. A, B, C = cumulus pieces surrounding oocytes that failed to cleave after exposure to sperm; D, E, F = cumulus pieces surrounding oocytes that developed into blastocysts after fertilization.

miRNAs were identified according to entries in miRBase Release 20. A mean of 332 miRNA species had more than 10 reads (Figure 2, blue bars), while 240 miRNA species had more than 50 reads per sample (Figure 2, red bars). The majority of the miRNAs were present in all samples; of the miRNA species identified, 276 with more than 10 counts and 205 with more than 50 counts were present in all samples. No repeatable differences in the numbers of miRNAs identified were present between the two types of sample (Figure 2).

In short, cumulus cells harvested from the COCs of oocytes that failed to cleave after exposure to sperm and those that yielded a blastocyst showed a similar expression of miRNAs. Of all of the identified miRNAs, miR-21-5p was the most abundant (>2,000,000 reads, Table 2), but the number of reads was similar between the two groups. Different members of the let-7 family, miR-155 and miR-99a-5p also gave more than 100,000 reads in each group (Table 2).

**Figure 3:**
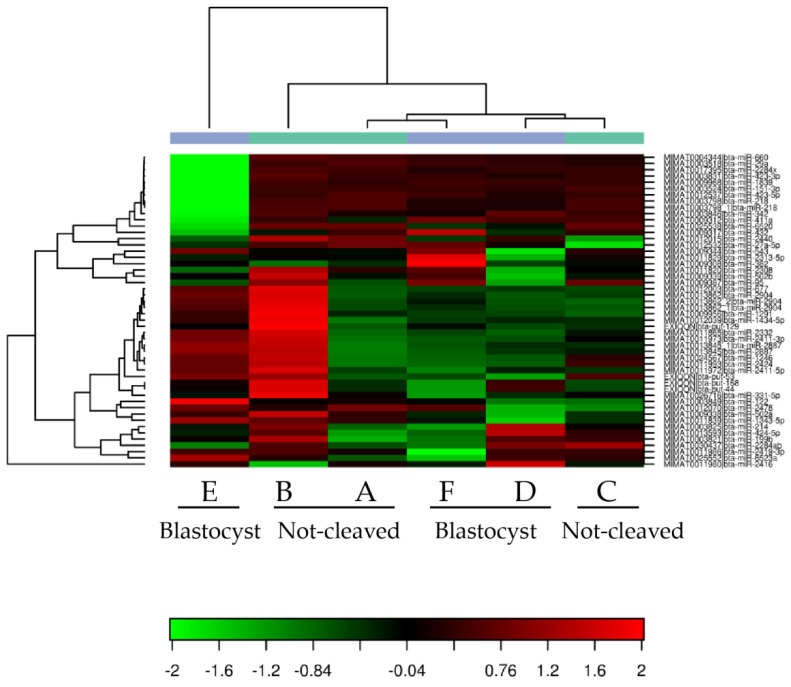
Heat map of the tested cumulus cell samples. Heat map and unsupervised hierarchical clustering by sample and miRNA. The clustering was performed on all samples and on the top 50 miRNAs with the highest % CV based on TPM (transcripts per million) normalized reads. A, B, C = cumulus pieces surrounding oocytes that failed to cleave after exposure to sperm; D, E, F = cumulus pieces surrounding oocytes that developed into blastocysts after fertilization.

**Figure 4:**
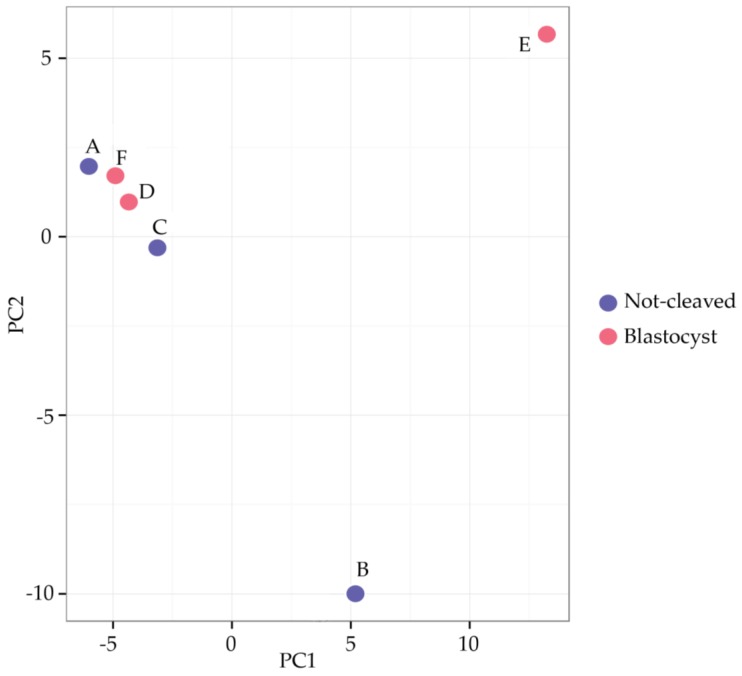
Principal component analysis (PCA) plot. The PCA was performed using the 50 miRNAs that had the largest variation across all samples. The features have been shifted to be zero centered (i.e., the mean value across samples was shifted to zero) and scaled to have unit variance (i.e., variance across samples was scaled to one) before the analysis. The groups do not cluster, not least because Samples B and E differ greatly from the rest for the primary component and from each other for the secondary component. Red circles = cumulus pieces surrounding oocytes that failed to cleave after exposure to sperm; blue circles = cumulus pieces surrounding oocytes that developed into blastocysts after fertilization.

When comparing the miRNAs expressed in cumulus cells from around an oocyte that developed into a blastocyst after fertilization with those surrounding an oocyte that had not cleaved, a high variability between the different pooled samples and no repeatable differences between the groups were observed (Figure 3). Samples A and B (cumulus cells enclosing oocytes that did not cleave after exposure to sperm) and Samples F and D (cumulus cells surrounding oocytes that developed into blastocysts) grouped together after hierarchical clustering. By contrast, Samples C (cumulus cells enclosing oocytes that did not cleave) and E (cumulus cells surrounding oocytes that developed into blastocysts) exhibited a markedly different miRNA expression profile; this was also apparent after principal component analysis (Figure 4). In general, within the tested groups, the expression of miRNAs was variable, which presumably contributed to the absence of differences in the expression profiles between the groups.

### 2.3. Quantitative Real-Time Reverse Transcription-PCR (qRT-PCR) from Pooled Cumulus Complex Samples

**Figure 5:**
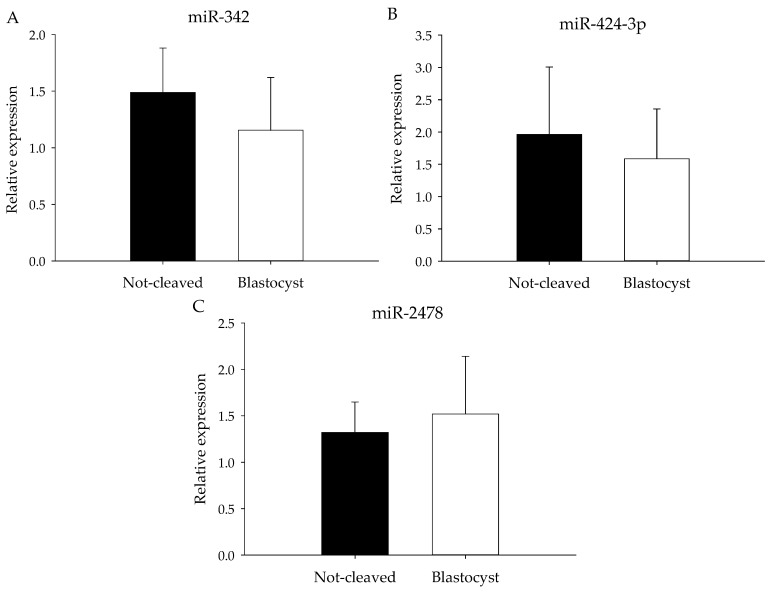
miRNA expression of pooled cumulus cell samples. Mean miRNA expression (qRT-PCR) for (**A**) miR-342, (**B**) miR-424-3p and (**C**) miR-2478 in pooled cumulus cell samples enclosing oocytes that failed to cleave (black bars) or that developed into blastocysts (white bars) after exposure to sperm. All samples were normalized for let-7a, miR-26a and miR-191 expression.

Although miRNA expression did not differ significantly between the two groups, expression of miRNAs for which the abundance appeared to differ according to the heat map (Figure 3) was further examined by qRT-PCR. The selected miRNAs had low or moderate total reads at sequencing, and to compensate for the anticipated low expression levels, pooled samples of cumulus cells from individually-cultured COCs were analyzed. The expression of miR-214, miR-424 and 2284ab was below the level of detection by qRT-PCR, and indeed, the read numbers for these miRNAs were around or below 100 (Table 3). For miR-342, miR-2478 and miR424-3p, expression levels (Table 3) were similar in the cumulus cells surrounding an oocyte that gave rise to a blastocyst to those in cumulus cells around oocytes that did not cleave (Figure 5).

### 2.4. qRT-PCR from Individual Cumulus Complex Samples

**Figure 6:**
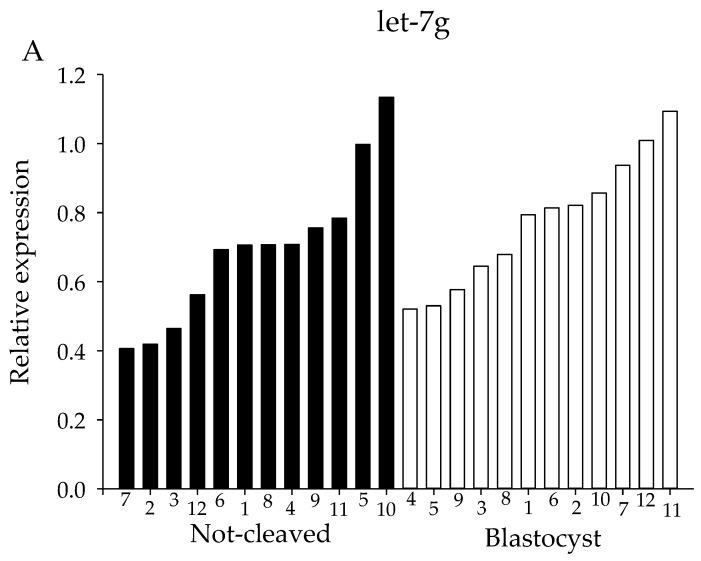

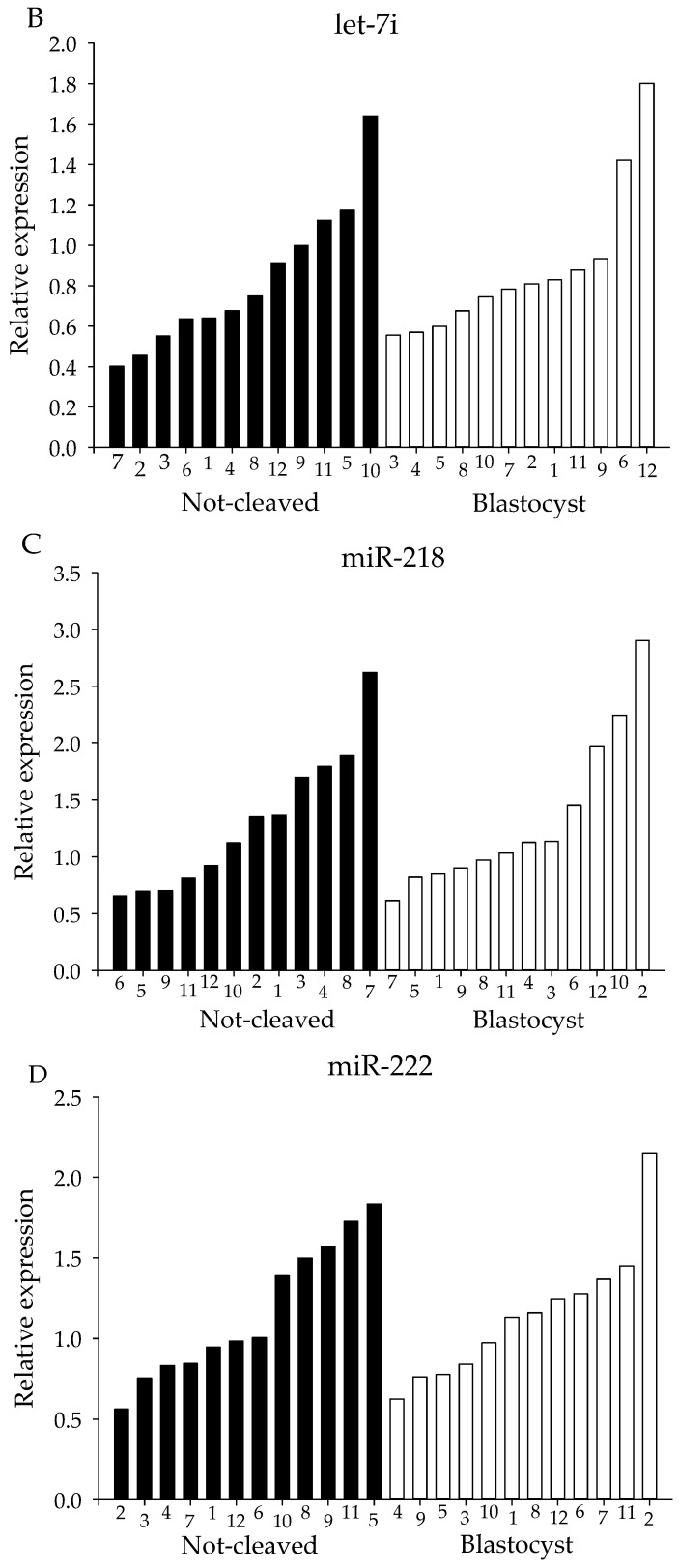
miRNA expression of individual miRNA samples. miRNA expression for cumulus cells from individual COCs. Expression levels of (**A**) let-7g, (**B**) let-7i, (**C**) miR-218 and (**D**) miR-222 as determined by qRT-PCR. X-axes depict individual cumulus complex samples from COCs that had enclosed an oocyte that either failed to cleave (black bars) or developed into a blastocyst (white bars) after exposure to sperm. All samples were normalized for expression of let-7a, miR-26a and miR-191.

For both the sequencing and the initial qRT-PCR analysis, pooled samples of cumulus cells from individually-cultured COCs were used. However, since oocyte quality is likely to be dependent on many different factors acting at different times and on different pathways, pooling of the samples might obscure miRNAs that are involved in regulating oocyte competence. Therefore, expression levels of candidate miRNAs were determined using qRT-PCR in cumulus cells from individual COCs. The selection of miRNAs examined was based on the sequencing results; four of the selected miRNAs had been highly expressed and showed a moderate difference in expression between ‘non-cleaved’ COCs and COCs that gave rise to a blastocyst, namely let-7g, let-7i, miR-222 and miR-218. For all four miRNAs, a high variability in miRNA expression between the twelve individual samples within a group was observed (Figure 6).

Finally, analysis of the sequencing data indicated 178 novel putative miRNAs. Not all of these miRNAs were detected in the cumulus cell samples; however, nine novel miRNA species were detected with a mean of >10 reads, indicating that the miRNAs are indeed generated and expressed (Table 4). However, the expression levels of these novel miRNAs did not differ between the non-cleaved and blastocyst groups.

## 3. Discussion

In this study, the miRNA expression profiles of cumulus cells from developmentally-competent COCs were compared with those from around oocytes did not cleave after exposure to sperm. As far as we know, this is the first study to examine whether cumulus cell miRNA expression can be used to predict the quality of oocyte before fertilization. In our study, samples of cumulus cells from individually-fertilized and cultured COCs were used and retrospectively assigned to post-fertilization developmental competence groups.

For identification of miRNAs expressed in cumulus cells, next generation sequencing was used. From both groups, three pooled samples of cumulus cells, each consisting of cells from around 10 COCs, were analyzed. Cumulus cells surrounding an oocyte that did not cleave after sperm exposure or developed into a blastocyst were chosen, to have the two most different stages to see a change in miRNA expression. However, no specific miRNA species were identified that could be correlated to oocyte quality, suggesting that miRNA expression in cumulus cells is not predictive of oocyte quality. Since our aim was to identify competent oocytes before fertilization, we decided to compare miRNA expression patterns in cumulus cells that did not cleave after exposure to sperm, versus those that developed into a blastocyst after exposure to sperm. This did not allow us to distinguish between those oocytes that were fertilizable and those that were not. For the identification of cumulus markers that would predict embryo developmental competence, comparison between cells originating from embryos that remained at the two-cell stage versus those that become a blastocyst would be useful, but this would always require fertilization and the generation of embryos. Oocyte fertilizability and developmental competence are dependent on various factors, and pooling of the cumulus pieces might have obscured any influence of individual miRNAs. In this respect, we further showed that the expression levels of several selected miRNAs varied greatly between cumulus complexes from individual COCs; at least for these miRNAs, the variation in expression was similar for competent and incompetent COCs. Oocytes from slaughterhouse ovaries were used in this study, which represent a heterogeneous population. Possibly, a more homogeneous oocyte population, for example originating from one breed, would reveal specific miRNAs. Our data indicate however that these could not be used as markers in a heterogeneous population, such as the human population.

A study of human oocytes reported a change in expression level between the germinal vesicle and the metaphase II stage for 15 of 722 miRNAs expressed during maturation [37], suggesting specific roles during maturation and early embryo development. It would be interesting to determine which miRNA expression levels change the most in bovine cumulus cell complexes during maturation. On a similar note, the difference in miRNA expression patterns between outer cumulus and inner cumulus, i.e., the cells furthest from or closest to the oocyte, might shed more light on the function of miRNA in cumulus cells.

Our sequencing results showed that miR-21-5p is the most abundant miRNA in bovine cumulus cells, followed by various members of the let-7 family and miR-155. In human cumulus cells, members of the let-7 family and miR-21 are also the most abundant miRNAs; however, their functions remain unclear [14]. An anti-apoptotic effect was proposed for miR-21 in mouse granulosa cells; however, it was not clear how this effect was achieved, because no change in apoptotic protein expression was observed after miR-21 knockdown [39]. A relatively low expression of miR-21 was detected in bovine oocytes, but the expression increased dramatically during early embryonic development [36]; similarly, a 25-fold increase in miR-21 expression was reported in pig cumulus cells during maturation [40]. A potential target of miR-21 is programmed cell death protein 4 (PDCD4), and interaction between miR-21 and PDCD4 has been reported in pig oocytes [40]. Whether miR-21 has a similar function in bovine cumulus cells remains to be determined.

Analyzing miRNA expression in oocytes and cumulus cells using a PCR array showed a negative fold change of miR-155 expression between bovine oocytes and cumulus cells, indicating higher expression in cumulus cells than in oocytes. Interestingly, this fold change increased during maturation, indicating that either the expression levels in the cumulus cells decreased or the level in the oocyte increased, or both [33]. miR-155 was also found to be abundantly expressed in exosomes recovered from bovine follicular fluid, and the expression level was higher in fluid from follicles that contained a growing oocyte compared to those with a fully-grown oocyte [41]. Based on the high expression levels of miR-155 in cumulus cells, it seems likely that this miRNA is produced by the cumulus cells themselves, although it cannot be excluded that cumulus cells take up miR-155 from the follicular fluid.

High expression levels of various let-7 family members have been reported in cumulus cells; among the most abundant let-7 family members were let-7f, let-7i and let-7g. Let-7 was also abundant in human and mouse cumulus cells [42]. Interestingly, let-7 miRNAs are negatively associated with pluripotency [43], although they are highly expressed in oocytes [35,44]. This indicates a high degree of tissue specificity of miRNA expression and underlines the likelihood that they are involved in regulating different processes at specific time points.

Although target prediction methodologies are improving, computational prediction of miRNA targets needs to be experimentally validated since predicted target sequences can give rise to false positives. In this respect, examination of candidate gene expression after overexpressing or downregulation of miRNAs appears essential, preferentially combined with a demonstration of an interaction between the miRNA and its target mRNA, for instance using luciferase reporter constructs [45].

Overall, the results of this study suggest that the quality of an oocyte within a COC before fertilization cannot be predicted by the miRNA expression palette of the cumulus cells. However, the high and dynamically changing expression levels of various miRNA species suggest that they are important for cumulus cell function. Whether cumulus cell miRNAs are transported into the oocyte remains to be investigated. The identification of novel miRNAs expressed by cumulus cells emphasizes that much remains to be discovered about these regulatory molecules.

## 4. Materials and Methods

All chemicals were purchased from Sigma-Aldrich (St. Louis, MO, USA) unless otherwise indicated.

### 4.1. Collection of Cumulus−Oocyte−Complexes, In Vitro Maturation and Fertilization

Cumulus−oocyte−complexes were collected and cultured as described previously [46]. COCs were matured for 23 h in groups of 60–70 in NaHCO_3_-buffered M199 (Gibco BRL, Paisley, U.K.) supplemented with 10% fetal bovine serum, 1% penicillin-streptomycin (Gibco BRL) and 0.05 IU follicle stimulating hormone/mL (Organon, Oss, The Netherlands) at 39 °C in a humidified atmosphere of 5% CO_2_-in-air. After maturation, small pieces of the cumulus complexes were retrieved using a narrow-bore Pasteur pipette. These cell clumps were transferred to lysis buffer (Exiqon, Vedbaek, Denmark) containing 10 µL/mL β-mercaptoethanol, labelled and stored at −80 °C until further use. Subsequent individual fertilization and embryo culture enabled retrospective linking of the cumulus pieces to the quality of the oocyte that they had enclosed.

In vitro fertilization was performed as described previously [47]. In short, Percoll-washed sperm cells from a bull of proven fertility were added to fertilization medium supplemented with 1.8 IU/mL heparin, 20 µM d-penicillamine, 10 µM hypotaurine and 1 µM epinephrine at a final concentration of 1 × 10^6^ sperm cells/mL. Terasaki microwell plates (Nalge Nunc International, Rochester, NY, USA) were used to allow single oocyte fertilization, and one oocyte per well was incubated in 10 µL of fertilization medium and covered with light mineral oil (Irvine Scientific, Santa Ana, CA, USA). After 18–22 h at 39 °C in a humidified atmosphere containing 5% CO_2_ and 7% O_2_, presumptive zygotes from single oocyte fertilization were denuded by repeated aspiration through a narrow pipette; presumptive zygotes from group culture were denuded by vortexing for 3 min; all denuded zygotes were transferred to synthetic oviductal fluid (SOF) for further culture [46]. On day 5 of culture, cleaved embryos were transferred to a new well containing fresh SOF and cultured until day 8.

### 4.2. RNA Isolation

Total RNA was isolated using the miCURY RNA isolation kit (Exiqon), according to the manufacturer’s protocol. In short, samples were lysed with 350 µL lysis buffer containing 10 µL/mL β-mercaptoethanol and shearing stress was applied by aspiration through a 23-gauge needle, followed by the addition of 200 µL 100% ethanol. The samples were loaded onto the columns provided, washed by centrifugation, and the RNA was eluted using 50 µL elution buffer.

An additional concentration step was performed on cumulus cell samples from individual COCs. To do this, the RNeasy MinElute Cleanup Kit (Qiagen, Hilden, Germany) was used, and the RNA was isolated as per the manufacturer’s instructions. In brief, 350 µL RLT buffer and 675 µL 100% ethanol were added to 100 µL of sample. This solution was transferred to an RNeasy MinElute spin column, and after three centrifugation and washing steps, the RNA was eluted using 17 µL RNase-free water.

### 4.3. Library Preparation and Next Generation Sequencing

Library preparation and next generation sequencing was performed by Exiqon. The concentration of total RNA from pooled cumulus samples (10–16 COCs) varied between 10 and 20 ng/µL. To obtain a minimum of 100 ng total RNA, 45 µL of each sample were converted into miRNA next generation sequencing libraries using a NEBNEXT kit (New England Biolabs, Ipswich, MA, USA) as per the manufacturer’s instructions. Adaptors were ligated to the ends of the sequences, and libraries were generated using 3′ and 5′ rapid amplification of cDNA ends (RACE)-like protocols. Libraries were purified after 15-cycle pre-PCR using QiaQuick columns (Qiagen) and size fractioned on a LabChip XT (Caliper, Hopkinton, MA, USA). Bands representing 15–40-bp fragments were excised with an automated gel cutter, evaluated using a Bioanalyzer DNA 2100 chip and quantified by qPCR. Samples were normalized and pooled in equimolar concentrations [48] and sequenced using v3 sequencing (Illumina Next Seq 500 system, San Diego, CA, USA). Eight-level quality score-binning was used, enabling a more compact storage of raw sequences [49].

The miRNA sequencing data have been deposited in NCBI’s Gene Expression Omnibus database and are accessible through Geo series Accession Number GSE94771.

### 4.4. First-Strand Synthesis and qRT-PCR

The universal cDNA synthesis kit II (Exiqon) was used to perform first-strand synthesis, with 10 µL template RNA in a total volume of 20 µL as per the manufacturer’s instructions. Samples were incubated for 1 h at 42 °C followed by 5 min at 95 °C and then immediately cooled on ice.

PCR was performed in a total volume of 15 µL with 1 µL cDNA, 1.5 µL locked nucleic acid (LNA) primer pair (Exiqon) and 7.5 µL PCR master mix (Bio-Rad, Hercules, CA, USA). Quantitative real-time PCR was performed using a CFX Connect™ Real-Time PCR detection system (Bio-Rad). The program started with 10 min at 95 °C followed by 40 cycles each of 10 s at 95 °C and 60 s at 60 °C. Melting curves were plotted after each cycle series.

**Figure A1:**
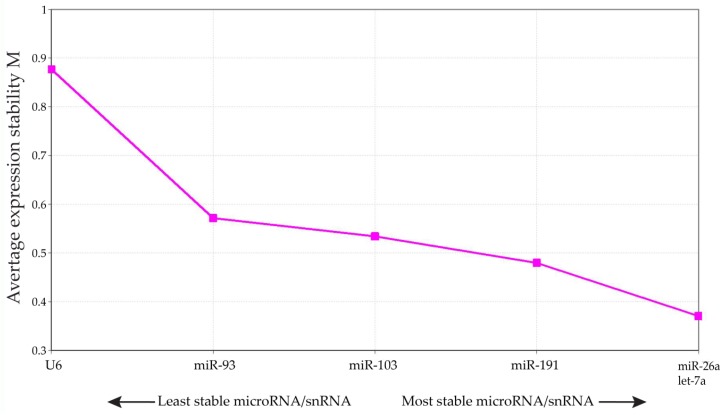
Average expression stability of different mi/snRNAs. Average expression stability (M) of miRNAs/snRNA in bovine cumulus cells. The three miRNAs with the lowest M-values were selected for qRT-PCR normalization.

A standard curve of a 3-fold dilution series was obtained by plotting the log of the starting amount against the cycle threshold value of the dilution series. Different potential reference miRNAs were tested, and in accordance with GeNorm guidelines [50], the three small RNAs with the most stable M-value were considered optimal and adequate for normalization (Appendix A: Figure A1). The geometric means of expression for the reference miRNAs miR-26a, miR-191 and let-7a were used for normalization of the target miRNAs.

### 4.5. Statistical Analysis

Comparisons between culture conditions and the resulting cleavage and blastocyst production rates were performed using Student’s *t*-tests. Threshold cycle (Ct)-values of possible reference miRNAs were analyzed using GeNorm [50].

## Figures and Tables

**Table 1:** Comparison of group and individual embryo culture. Comparison of cleavage and further development of bovine cumulus−oocyte−complexes (COCs)/zygotes cultured in groups or individually.

			Day 5			Day 8
**Culture type**		Total	Not–cleaved	2 to 8	>8	Blastocyst
Group	n	481	121	207	153	129
	%		25.2	43	31.8	26.8
Individual	n	188	35	86	67	53
	%		18.6	45.7	35.6	28.2

**Table 2:** Most abundantly-expressed microRNAs (miRNA).

miRNA	Counts NC	Counts Blast
bta-miR-21-5p	2,324,236	2,915,237
bta-let-7f	431,569	335,113
bta-let-7i	302,000	256,981
bta-let-7g	228,393	177,529
bta-miR-155	225,179	158,161
bta-let-7a-5p	217,033	158,238
bta-miR-99a-5p	129,482	112,348
bta-miR-30d	85,620	75,966
bta-miR-26a	73,820	56,991
bta-miR-320a	59,660	53,015
bta-miR-92a	55,108	47,255
bta-miR-10b	54,473	66,439
bta-miR-202	38,887	32,030
bta-miR-148a	38,574	40,607
bta-let-7b	36,653	29,117
bta-miR-532	34,632	32,815
bta-miR-99b	29,386	31,287
bta-let-7e	25,088	20,497
bta-miR-125a	22,159	20,436
bta-let-7c	23,668	17,542

The list of the 20 most abundantly-expressed miRNAs in bovine cumulus cells. NC = cumulus pieces enclosing oocytes that failed to cleave after exposure to sperm; Blast = cumulus pieces enclosing oocytes that developed into a blastocyst after fertilization.

**Table 3:** Sequencing counts of chosen miRNAs.

miRNA	Not-cleaved			Blastocyst		
	A	B	C	D	E	F
bta–miR–214	35	33	30	119	22	13
bta–miR–218	4,546	1,451	2,198	3,004	86	1,411
bta–miR–222	3,228	1,175	2,090	1,724	1,291	1,151
bta–miR–342	1,267	802	963	2,856	20	515
bta–miR–424–3p	354	62	166	159	208	181
bta–miR–424–5p	27	26	34	113	20	16
bta–miR–2284ab	32	20	41	70	12	13
bta–miR–2478	357	92	66	105	154	150
bta–let–7i	428,545	199,640	277,815	396,881	225,420	148,643
bta–let–7g	332,160	154,890	198,131	237,141	163,940	131,506

Sequence counts for miRNAs examined further. Not cleaved = cumulus pieces surrounding oocytes that failed to cleave (Samples A, B, C); Blastocyst = cumulus pieces surrounding oocytes that developed into blastocysts after fertilization (Samples D, E, F).

**Table 4:** Novel expressed miRNAs, predicted and expressed miRNAs identified from read data based on read count and secondary structure according to the miRPara (miRNA prediction software tool) classification score. The description of chromosomal location is shown with Chr (chromosome), start and stop.

Name	Chr	START	STOP	SEQUENCE
bta-put-45	chr7	12981779	12981801	CCGUGCCUACUGAGCUGAAACAC
bta-put-129	chr21	69641635	69641655	UGCAAGCAACACUCUGUGGCA
bta-put-53	chr7	53516556	53516579	UAUACUCUGAUUGGUUCAUUAUGA
bta-put-79	chr29	1067001	1067020	AUGGUCAUUACCAAGGCUUU
bta-put-168	chr7	5206807	5206828	UCAAAGUGAAUUUGGAGGUUCU
bta-put-44	chr7	5206802	5206823	UCAAAGUGAAUUUGGAGGUUCU
bta-put-82	chr29	41693966	41693989	GAUCCGCGUAAUGUACGGAGGUAG
bta-put-156	chr5	103546363	103546386	GGACCUCAGUUCCAAACCUCUGCC
bta-put-25	chr5	104238656	104238676	AUGUGGACCCAGGGAGCUGGG
